# Nanoparticles prepared from pterostilbene reduce blood glucose and improve diabetes complications

**DOI:** 10.1186/s12951-021-00928-y

**Published:** 2021-06-27

**Authors:** Xi Zhao, Anhua Shi, Qiong Ma, Xueyan Yan, Ligong Bian, Pengyue Zhang, Junzi Wu

**Affiliations:** 1 Yunnan Provincial Key Laboratory of Molecular Biology for Sinomedicine, School of Basic Medical, Yunnan University of Chinese Medicine, Kunming, Yunnan 650500 P.R. China; 2 College of Clinical Medical, Kunming Medical University, Kunming, Yunnan 650500 PR China; 3 College of Basic Medicine, Kunming Medical University, Kunming, Yunnan 650500 PR China; 4 Key Laboratory of Acupuncture and Tuina for Treatment of Encephalopathy, College of Acupuncture, Tuina and Rehabilitation, Yunnan University of Traditional Chinese Medicine, Kunming, 650500 China

**Keywords:** 3-acrylamidophenylboronic acid (AAPBA), Diabetes complications, Diabetes mellitus, Insulin delivery, Nano-carrier, Pterostilbene (PTE)

## Abstract

**Background:**

Diabetes complications are the leading cause of mortality in diabetic patients. The common complications are decline in antioxidant capacity and the onset of micro-inflammation syndrome. At present, glucose-responsive nanoparticles are widely used, as they can release insulin-loaded ultrafine particles intelligently and effectively reduce blood sugar. However, the toxicology of this method has not been fully elucidated. The plant extracts of pterostilbene (PTE) have a wide range of biological applications, such as antioxidation and inflammatory response improvement. Therefore, we have proposed new ideas for the cross application of plant extracts and biomaterials, especially as part of a hypoglycaemic nano-drug delivery system.

**Results:**

Based on the PTE, we successfully synthesised poly(3-acrylamidophenyl boric acid-b-pterostilbene) (p[AAPBA-b-PTE]) nanoparticles (NPs). The NPs were round in shape and ranged between 150 and 250 nm in size. The NPs possessed good pH and glucose sensitivity. The entrapment efficiency (EE) of insulin-loaded NPs was approximately 56%, and the drug loading (LC) capacity was approximately 13%. The highest release of insulin was 70%, and the highest release of PTE was 85%. Meanwhile, the insulin could undergo self-regulation according to changes in the glucose concentration, thus achieving an effective, sustained release. Both in vivo and in vitro experiments showed that the NPs were safe and nontoxic. Under normal physiological conditions, NPs were completely degraded within 40 days. Fourteen days after mice were injected with p(AAPBA-b-PTE) NPs, there were no obvious abnormalities in the heart, liver, spleen, lung, or kidney. Moreover, NPs effectively reduced blood glucose, improved antioxidant capacity and reversed micro-inflammation in mice.

**Conclusions:**

p(AAPBA-b-PTE) NPs were successfully prepared using PTE as raw material and effectively reduced blood glucose, improved antioxidant capacity and reduced the inflammatory response. This novel preparation can enable new combinations of plant extracts and biomaterials to adiministered through NPs or other dosage forms in order to regulate and treat diseases.

**Graphic abstract:**

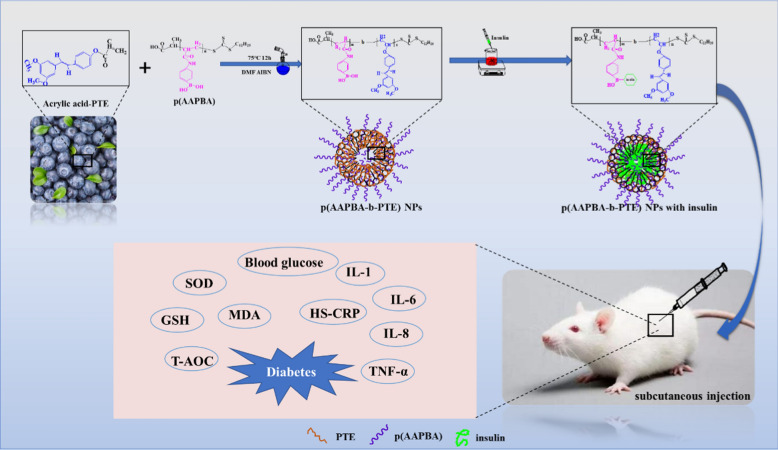

**Supplementary Information:**

The online version contains supplementary material available at 10.1186/s12951-021-00928-y.

## Background

Diabetes (diabetes mellitus, DM) is a metabolic disease that is characterised by high blood glucose, which is caused by dysfunction in insulin secretion and/or insulin resistance [1]. It causes damage or defects in various tissues, especially the eye, kidneys, heart, blood vessels and chronic nerves. Diabetes complications such as cardiovascular disease are major causes of mortality. Glycaemic instability and a long-term microinflammatory state are two central factors in the development of diabetes complications [2]. At present, the most direct and effective way to treat diabetes is to supplement deficient insulin creation with exogenous insulin. However, daily injection of insulin reduces patient adherence to insulin therapy and reduces their quality of life. Insufficient or excessive insulin injections and/or irregular use of medications can cause blood glucose instability [3]. Moreover, many diabetic patients experience long-term low antioxidant capacity and micro-inflammation [4]. The decrease in antioxidant capacity is determined by decreases in superoxide dismutase (SOD) and glutathione (GSH) in the blood, whereas the microinflammatory state refers to an increase in hypersensitive C-reactive protein (Hs-CRP), interleukin-1 (IL-1) and tumour necrosis factor-α (TNF-α). The decrease or increase of these markers can predict the occurrence of cardiovascular events and the prognosis of diabetes complications. Therefore, it is necessary to improve the antioxidant capacity and long-term microinflammatory status of diabetic patients. An approach to achieving this aim is to discover ways to modify the insulin pharmacokinetics, thus making its onset faster or its effect longer lasting and closer to the human pathophysiological state.

Phenylboronic acid (PBA) has always been a research hotspot as a potential mechanism for controlling blood sugar stability [5, 6]. The phenylboronic acid group can be reversibly combined with sugar compounds containing cis-diol chains [7]. Under a mutual repulsion interaction of the same charge in the molecule, polymers of the reticular carrier structure undergo different degrees of swelling or damage and then release the corresponding encapsulated drugs [7]. Phenylboronic acid-based nanocarriers can not only achieve effective sustained release of insulin but also can regulate their drug release rate according to the glucose concentration in the body [8]. For this reason, increasingly more studies have been devoted to developing glucose-responsive drug release systems. The normal pH of the human physiological environment is 7.4, while the pKa range of PBA and its derivatives is generally 8.2–8.6 [9]. The pKa of the glucose-responsive polymer based on phenylboronic acid is higher than the physiological pH. Therefore, when phenylboronic acid groups in the polymer are not in the ionised state, it is difficult for the polymer to react to glucose. Therefore, a prerequisite for the application of a phenylboronic acid drug carrier in the treatment of diabetes is to reduce its pKa so that it can achieve a glucose-sensitive performance within the physiological environment. To achieve this aim, researchers have tried different methods. For example, Matsumoto [10] proposed a molecular strategy to operate an insulin delivery system that is self-regulated under normal physiological conditions (pH 7.4, 37 °C), including the use of a novel phenylboronic acid derivative (4-[1,6-dioxo-2,5-diaza-7-oxamyl] phenylboronic acid: DDOPBA), which has a rather low pKa (∼ 7.8), and the adoption of poly(N-isopropylmethacrylamide) (PNIPMAAm) for the main chain. Zheng [11] et al. also prepared an amphiphilic glycopolymer (p[LAMA-r-AAPBA]), which can be delivered with lower blood glucose through the nasal cavity. In previous studies, we used N-vinylcaprolactam (NVCL), diethylene glycol dimethacrylate (DEGMA) and 6-O-vinylazeloyl-d-galactose (OVZG) to copolymerise with AAPBA, with which a variety of glucose-sensitive carriers were successfully synthesised [12–14]. In a summary of previous studies, various formations of polymers, including thermosensitive monomers, pH-sensitive monomers, polyaminoacids, and glycolipid monomers, can be made glucose responsive through various processing methods (Summarised in Additional file 1: Table S1). However, neither the degradation time nor process has been fully elucidated, especially for the functional monomer. Biomaterials can develop chronic and long-term toxicity during the in vivo degradation process, which may cause secondary damage to the human body; therefore, further research and improvement of glucose-sensitive carriers are required [15].

With continuous exploration of the development and application of traditional Chinese medicine, natural plant extracts have gradually attracted people’s attention. It was discovered that many plant extracts improve antioxidant capacity and anti-inflammatory effects. Pterostilbene (PTE; trans-3,5-dimethoxy-4′-hydroxystilbene), a trans-stilbene compound, is a methylated derivative of resveratrol which has a higher, more stable bioavailability than resveratrol [16]. PTE has a variety of biological activities, such as lowering blood lipids and blood glucose, fungi inhibition, antioxidation and anti-tumorigenesis [17, 18]. In addition, it has a variety of preventive and therapeutic effects on neurological diseases, cardiovascular diseases, metabolic diseases and blood diseases [19–21]. An experimental study by Tastekin [22] found that the blood glucose, serum insulin and malondialdehyde (MDA) levels were close to normal after PTE administration in diabetic rats, and the rats showed better morphological and structural enhancement of skeletal muscle. Kosuru [23] et al. administered PTE treatment to fructose-induced diabetic rats and found that it successfully improved blood sugar control and insulin sensitivity, as well as reduced metabolic disorders and liver oxidative stress. PTE improved antioxidant capacity and reduced the microinflammatory response [24]. For example, PTE activates nuclear factor-2, which can cause a high expression of heme oxygenase-1 and glutathione reductase, thus playing an antioxidative and anticancerous role [25]. In addition, PTE can activate protein kinase C reduced coenzyme II oxidase, thereby stimulating neutrophils to produce superoxide anions and peroxidase, which in turn decreased the expression of related inflammatory factors [26]. As these activities can improve the condition of diabetic patients, PTE has become a research focus for its potential to reduce blood glucose and treat diabetes complications. However, Lin [27] et al. conducted pharmacokinetic studies on standard deviation (SD) rats by using intravenous injection and oral administration of PTE and found that the half-life and clearance rate of intravenous PTE were (96.6 ± 23.7) min and (37.0 ± 2.5) min, respectively, and that the bioavailability of PTE by oral administration was greatly reduced. The reason may be that the first pass effect reduces the blood content of PTE. In addition, most plant extracts like PTE are easily oxidised, have poor water solubility, and low oral bioavailability, which limits their application. Many scholars have tried to improve the bioavailability of PTE by altering the dosage form or adjusting the production process to fully exploit the pharmacological activity of PTE for its potential to treat human diseases.

Here, we hypothesised that PTE could be esterified into a high molecular material and copolymerised with AAPBA to form a glucose-responsive polymer. To test this hypothesis, insulin was entrapped in polymers to prepare a batch of drug carriers that could release insulin intelligently. The performance, toxicology and therapeutics of the glucose-responsive polymer was investigated in the development of a batch of safe and nontoxic glucose-responsive drug carriers. Our research examined whether the glucose-responsive drug carriers could intelligently release insulin, and whether the pharmacological activity of PTE could be released when the polymer degraded. Furthermore, we investigated whether these capabilities stabilised blood glucose levels, improved antioxidant capacity and/or reversed the microinflammatory state. We aimed to demonstrate the strong natural activity of plant extracts and their potential to be exploited for the development of numerous safe, nontoxic and effective copolymers. Considering the pharmacological activity and characteristics of PTE, plant extracts have the potential to reduce unknown toxicities of biomaterials to improve their adaptability for medical applications in the human body.

## Materials and methods

### Materials

Esterified PTE was synthesised by Hangzhou Yu Hao Chemical Industry; AAPBA was synthesised by Wuhan Jusheng Technology Co., Ltd.; 2, 2-azobisisobutyronitrile (AIBN) was purchased from Sigma Aldrich (Shanghai, China); insulin (27 U.mg ^−1^) was purchased from Shanghai Macklin Biochemical Technology Co., Ltd. (Shanghai, China); and dimethyl sulfoxide, diethyl ether and methanol and other analytical pure solvents were purchased from Sinopharm Chemical Reagent Co., Ltd. for used in storage. Human normal liver L0_2_ cells and human hepatoma SMMC-7721 cells were purchased from Shanghai Jia YUAN Biological Co., Ltd. (Shanghai, China); an (3-[4,5-dimethylthiazol-2yl]-2,5-diphenyl-tetrazolium bromide) (MTT) kit was purchased from Shanghai Enzyme Technology Co., Ltd. (Shanghai, China); ultrapure water was obtained from home-made in the laboratory.

### Preparation of P(AAPBA-b-PTE)

**Scheme 1:**
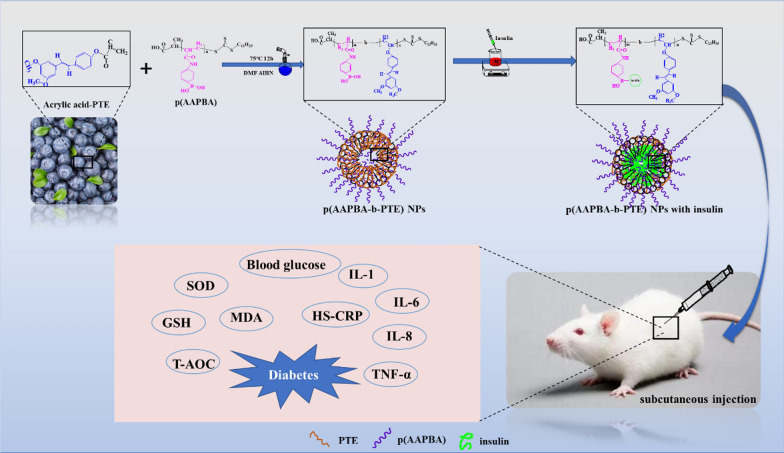
Synthesis process of p(AAPBA-b-PTE) and p(AAPBA-b-PTE) NPs loaded with insulin, and p(AAPBA-b-PTE) NPs loaded with insulin can reduced blood glucose, improved antioxidant capacity and reversed micro-inflammation by subcutaneous injection

**Scheme 2:**
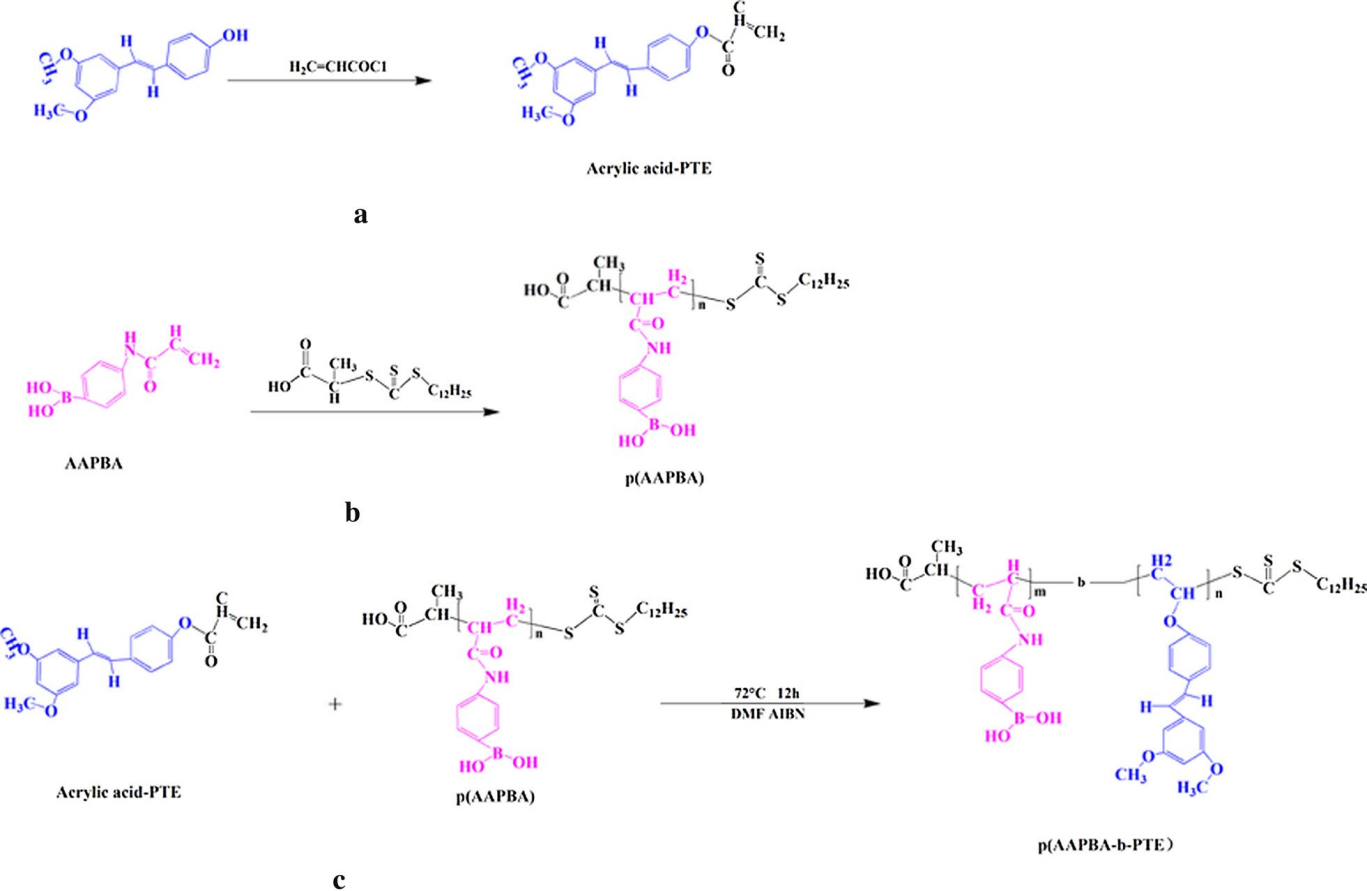
Synthesis of molecular structure of acrylic acid-PTE (**a**), p(AAPBA) (**b**) and p(AAPBA-b-PTE) (**c**)

The first step involved preparing acrylic acid-PTE by using acryloyl chloride to endow the C=C double bond of pterostilbene (Scheme 2a), so that it could be used as a component in the polymer materials. P(AAPBA) was prepared according to our previously published protocol [12]. Next, as shown in Scheme 1C, p(AAPBA-b-PTE) was synthesised via ‘one-pot’, as reported in our previous literature [13]. In the first step, p(AAPBA) and acrylic esterified PTE were used as reaction monomers (Scheme 2b) and AIBN was used as the initiator (proportion shown in Table 1), which were mixed in dimethylformamide (DMF) and water (DMF: water = 9:1 ml) and sealed in a 50 ml round bottom flask. The second step involved the use of a vacuum pump to pump out the air and nitrogen until the bubbles disappeared. Then, nitrogen was added to the flask, which was repeated in three cycles, and the flask was placed in an oil bath for 12 h at 70 °C and stirred at a rotating speed of 20 r/min (Scheme 2c). Finally, the reaction flask was placed in ice water to stop the polymerization; the solution was precipitated three times in ether, filtered and dried in a vacuum for 24 h. Finally, p(AAPBA-b-PTE) was obtained.

**Table 1:** Study the polymerisation reaction of different ratios (AAPBA, PTE) and AIBN

Samples	p(AAPBA)	PTE (mg)	AIBN (mg)	Yield (%)
p(AAPBA-b-PTE)1	1000	500	1.5	79.4
p(AAPBA-b-PTE)2	1000	100	1.2	80.2
p(AAPBA-b-PTE)3	1000	50	1.1	77.1

The yield was calculated using the final collected polymer amount/the input material amount

### Characterisation of p(AAPBA-b-PTE)

#### Proton nuclear magnetic resonance (^1^H NMR)

Samples weighing 5 mg were tested in a nuclear magnetic tube containing a deuterium solvent consisting of water + deuterium with sodium hydroxide, which was vortexed for 1 min to mix the sample thoroughly. Then, 5 mL was added to the NMR tube, tested and analysed with an Avance400 Nuclear Magnetic Resonance Spectrometer (Japan Electronics Co., Ltd., Beijing, China), and the results were analysed with MestReNova software.

#### Fourier transform infrared spectroscopy (FTIR)

Samples weighing 5 mg were dried in a vacuum and grinded into powder. The scanning method was used for infrared measurement; the scanning range was 400–4,000 cm^−1^; and the scanning interval was 4 cm^−1^. The FTIR samples were collected and recorded with spectral data, and their chemical structures were deduced according to their characteristic absorption peaks. A Thermo Scientific Nicolet 5 FTIR Spectrometer (Thermo Fisher Scientific, Hercules, CA, USA) was used, and the results were analysed by OriginPro 9.0 software.

#### Thermogravimetry (TG) or thermogravimetric analysis (TGA)

The 5 mg samples were tested in a small dry spot under the condition of nitrogen at a rate of 10 °C/ minutes, and the results were detected using a thermogravimetric analyser (TGA5500, TA Instruments Co., Ltd., Newcastle, DE, USA). The results were carried out using OriginPro 9.0 analysis software.

#### Gel permeation chromatography

The 5 mg samples were dissolved in 25 ml tetrahydrofuran solution and the molecular weight (Mw, Mn) and molecular weight distribution of the sample was detected using a gel permeation chromatography instrument (Waters LLC, Milford, MA, USA) at a flow rate of 1.0 mL/min and temperature of 35 °C.

#### Dynamic light scattering (DLS)

The 5 mg sample was weighed and dissolved in distilled water to prepare a solution with a concentration of 1 mg/ml. The hydrodynamic diameter (Dh [Particle size]) and polydispersity index (PDI) of the NPs in water were measured using DLS. The results were expressed as the mean ± SD. (n = 3).

### Preparation of p(AAPBA-b-PTE) NPs

First, 5 mg of the p(AAPBA-b-PTE) was dissolved in a mixed solution of 2 mL dimethyl sulfoxide (DMSO) and water (1:1v/v), which was then slowly added at 600 rpm to 20 mL pure water, in which a small magnet it was placed. After 3 h of ice water bath at 4 °C, the suspension was centrifuged at 12,000 rpm in a low-temperature, high-speed centrifuge for 10 min. Then, the suspension was dispersed into ultrapure water (10 mL), transferred to a dialysis tube (MWCO6000) for 72-h dialysis at room temperature, and the water was changed every 4 h during dialysis to remove organic solvents. Then, the protein solution was frozen in a − 79 °C refrigerator for 12 h, then freeze-dried in a − 55 °C in a vacuum freeze-dryer to obtain p(AAPBA-b-PTE) NPs without insulin.

### Properties of p(AAPBA-b-PTE) NPs

#### Transmission electron microscopy (TEM)

Under 38 °C temperature, 1 mg/mL samples of the suspension of were added, one by one, to the copper net covered with a Formvar carbon film with an eyedropper. After the solvent was completely evaporated, the sample was dried. A transmission electron microscope (JEM-2100, JEOL, Japan) was used to select different areas for signal acquisition.

#### Zeta potential

To determine the zeta potential, 0.4 mg samples were dispersed in 1 mL deionised water, then analysed using a Zeta Potential Analyser (ZetaPlus/90Plus, Brookhaven Instruments Corporation, New York, NY, USA).

#### pH sensitivity test

The 4 mg samples were placed in PBS (10 mL) at different pH levels (5.5, 6.0, 6.5, 7.0, 7.5, 8.0, 8.5, 9.0, 9.5, or 10.0), and the particle size distribution of the samples in the aqueous phase was measured by DLS nanoparticle size analyser (Zetasizer Nano S, Malvern Instruments, Malvern, England, UK).

#### Temperature sensitivity test

The 4 mg NPs were dissolved in 10 mL PBS aqueous solution with a pH of 7.4, and the temperature was controlled at 10 °C, 15 °C, 20 °C, 25 °C, 30 °C, 35 °C, 40 °C or 45 °C. DLS was used to detect changes in particle size.

#### Glucose sensitivity test

The samples were dissolved in a 10 mL pH aqueous solution of 7.4 PBS, then the glucose concentration of the PBS solution was adjusted to − 0.5, 0, 0.5, 1, 1.5, 2, 2.5, 3 or 3.5 g/L, and the particle size was detected using DLS.

#### Elasticity test

The sample was added to 10 mL PBS aqueous solution with a pH value of 7.4, and then the glucose concentration of PBS solution was adjusted to 0, 3, 0, 3, 0, 3, 0, or 3 g/L. The hydrodynamic diameter was measured using DLS.

### Preparation of p(AAPBA-b-PTE) NPs containing insulin

The preparation method for insulin-encapsulated p(AAPBA-b-PTE) NPs is basically the same as that of insulin-free p(AAPBA-b-PTE) NPs. The difference is that a certain amount of insulin is weighed in advance, and 50 mg p(AAPBA-b-PTE) is dissolved in a mixed solvent of 1:1 v/v of DMSO to 2 mL water volume. The follow-up operation was consistent with the preparation of insulin-free NPs.

#### Detection of drug loading and the sealing rate

After the p(AAPBA-b-PTE) NPs containing insulin were obtained, they were centrifuged at 12,000 rpm and washed with water three times. The drug loading and entrapment efficiency (EE) of insulin were determined using the bicinchoninic acid (BCA) method, and the amount of free insulin in the supernatant after the BCA reaction was measured using the Bradford method with an ultraviolet spectrophotometer (Shimadzu UV2550) at 562 nm. The entrapment efficiency (EE) and drug loading (LC) of NPs were calculated using the following formulae:

$${\text{EE }} = {\text{ }}\left( {{\text{total insulin mass }} - {\text{ free insulin mass}}} \right){\text{/total insulin mass }} \times {\text{ 1}}00\% ;$$

$${\text{LC }} = {\text{ }}\left( {{\text{total insulin mass }} - {\text{ free insulin mass}}} \right){\text{/mass of NPs }} \times {\text{ 1}}00\%$$

All measurements were repeated three times and averaged.

#### Insulin release test

To test the efficiency of insulin release, four samples of 5 mg loaded insulin p(AAPBA-b-PTE) NPs were dispersed in 20 mL PBS (0.1 m) aqueous solution with a pH of 7.4 and a temperature of 37 °C. The glucose concentrations of the four aqueous solution were 0, 1, 2 and 3 mg/mL, respectively. Under the condition of 100 rpm oscillation, the drug release was determined at a fixed time point. When preparing the samples, the supernatant of 1 mL was taken out with a liquid transfer gun, and then a fresh preheating buffer was added (without insulin). After that, the content of free insulin was detected by an ultraviolet spectrophotometer and BCA reagent under 562 nm.

#### PTE release test

The chromatographic column used for the PTE release test was an X select HSS T3 column (4.6 mm × 250 mm, 5 μm); the flow rate was 0.8 ml/min; the mobile phase was acetonitrile water (60:40); the detection wavelength was 306 nm and the injection volume was 10 μL. A mixture of 20 mg PTE with 200 ml distilled water was dispersed and dissolved with an ultrasonic wave, and the volume of the release medium was fixed as the mother liquor. The mother solution into the buffer solution, and six different concentrations of PTE solutions (10, 20, 30, 40, 60, 80 μ g.ml^−1^) respectively, were prepared. The absorbance was determined at 306 nm, and the drug release curve of the carrier was obtained. The standard curve of PTE was drawn according to the relationship between the concentration and the absorbance, and the standard curve equation (y = 80831x + 51,327, *R*^*2*^ = 1.0000) was established to calculate the drug release rate.

#### Examination of degradation

p(AAPBA-b-PTE) NPs weighing 5 mg were ultrasonically dispersed in a conical flask filled with 10 mL standard buffer solution (pH = 7.4), which was placed in a constant temperature oscillator. Each sample was centrifuged at regular intervals at 37 °C and freeze-dried to obtain the residues. A transmission electron microscope (JEM-2100, JEOL, Japan) was used to select different areas for signal observations of the morphological characteristics.

### Cell viability

The survival rate of p(AAPBA-b-PTE) NPs was evaluated by using human normal liver L0_2_ cells and human hepatoma SMMC-7721 cells. An MTT reagent was purchased from Promega company, and the experimental method was performed according to the manufacturer’s instructions. SMMC-7721 cells in the logarithmic growth phase were digested and separated with 0.25% trypsin and then re-suspended in RP-mi-1640 medium containing 10% foetal bovine serum. The number of cells was adjusted to 5 × 10^4^ cells/ml, and 200 μl per well was added to the 96-well culture plate. L02 cells were cultured in a Dulbecco's modified Eagle’s medium. Cells containing 20% foetal bovine serum were cultured in a humid environment containing 5% CO_2_ at 37 °C. Then, different concentrations of p(AAPBA-b-PTE) NPs were added to the plate for incubation. After 24 h, 40 μl of 5 µg/ml MTT solution was added to each well, and the culture was continued at 37 °C for 4 h. The supernatant culture medium in each well was sucked and discarded, and 300 μl DMSO was added to each well. The absorbance value (A) at 570 nm was detected with an enzyme reader (BS-1101, Huatai hehe [Beijing] Trading Co., Ltd., China). The cell viability was determined using the following formula: cell survival rate (%) = (average a value of experimental group/average a value of blank control group) × 100% (n = 3).

### Animal toxicology study

Twenty-four Kunming mice (19–23 g, half male and half female) were randomly divided into four groups. All animal experiments were approved by the Animal Use and Ethics Committee of Yunnan University of Chinese Medicine. In addition, all the mice used in this experiment were purchased from the Department of Experimental Zoology, Kunming Medical University, with Animal Qualification Certificate No. SYXK (Dian) K2020-0006.

The mice in each experimental group were intra-peritoneally injected with p(AAPBA-b-PTE)2 NPs of 10, 50 or 100 mg/kg/day. The control group was injected with normal saline of 1 ml/kg/day. Two weeks later, blood was taken from the Laboratory Department of the First People's Hospital of Yunnan Province to measure the red blood count (RBC), white blood count (WBC), mean cell volume (MCV), haematocrit (HCT) and other blood routine indexes, which were detected using an automatic biochemical instrument.

### Experimental design

Twenty-four Kunming mice (19–23 g, half male and half female) were reared in an environment-controlled room (temperature: 25 ± 2 °C, humidity: 55 ± 5%, and 12 h light–dark cycle). A hyperglycaemia model was induced with a high-fat and high-sugar diet for 2 months and intra-peritoneal injection of streptozotocin (STZ) [28]. The successful standard of the model was a fasting blood glucose ≥ 11.1 mmol/L and symptoms of polydipsia and polyuria [29]. After successful establishment of the model, the mice were randomly divided into three groups: the model group (n = 6), insulin injection treated group (n = 6) and p(AAPBA-b-PTE)2 group (n = 6). In addition, six healthy mice were selected as a normal group. After grouping the mice, a corresponding treatment was given. During treatment, the p(AAPBA-b-PTE)2 group was administered a single injection of p(AAPBA-b-PTE) nano-injection preparation coated with insulin (the injection volume was determined according to the simulated drug release in vitro), and the insulin-treated group was given an insulin solution injection (0.16 mg/day, 1 mg insulin was dissolved in sodium acetate solution of 0.05 mL). The model group and normal group were given a normal saline injection (0.05 ml/day). The general condition and subcutaneous injection were observed daily. The blood of the mice in each group at corresponding time points was collected from the tail vein, and the glucose level was determined using a glucometer (GT-1640; Guilin Renke Medical Technology Development Co., Ltd, Guilin, China). Two weeks later, all mice were anaesthetised with chloral hydrate and killed. The skin and main organs (heart, liver, spleen, lung and kidney) were collected, and staining was used to evaluate the morphology. The activity of malondialdehyde (MDA), total antioxidative capacity (T-AOC), glutathione (GSH) and serum SOD in serum was detected with a commercial reagent box (Nanjing Institute of Construction Biology Engineering), and the expression characteristics of Hs-CRP, IL-1, IL-6, IL-8 and TNF-a were detected using an enzyme-linked immunosorbent assay (ELISA Kit, Wuhan Doctoral Biotechnology).

### Statistical analysis

The measurement data were expressed as the mean ± SD. SPSS 23.0 software was used for statistical analysis. One-way analysis of variance was used to compare the mean between groups, and the least significant difference was used to compare the mean between groups. The difference was statistically significant (*p* < 0.05).

## Results and discussion

### Characterisation of p(AAPBA-b-PTE)

**Fig. 1.**
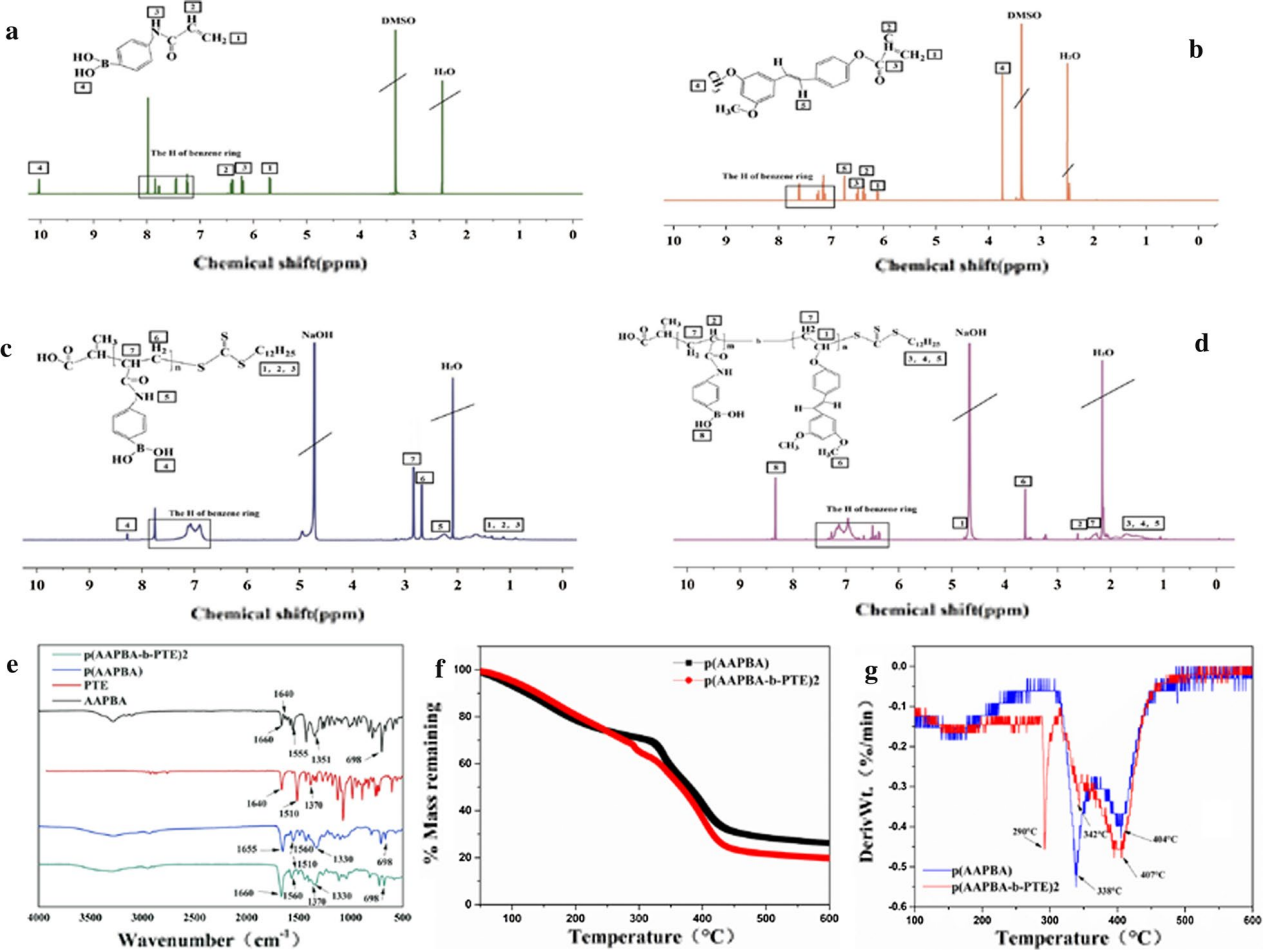
^1^H-NMR spectra results of AAPBA (**a**); PTE (**b**); p(AAPBA) (**c**); p(AAPBA-b-PTE) (**d**). **e** FT-IR spectra results. Thermal analysis of the polymers: DTG (**f**) and TG (**g**)

In our study, the structure of polymer p(AAPBA-b-PTE) was analysed using ^1^H nuclear magnetic resonance spectroscopy and Fourier transform infrared transmission spectroscopy (FTIR). As shown in Fig. 1a–d, we detected the ^1^H NMR spectra of AAPBA, PTE, p(AAPBA) and p(AAPBA-b-PTE)2 and their infrared peaks, which clearly indicated that polymerisation was successful.

The ^1^H-NMR spectrum of Fig. 1a–d exhibit individual peaks and peak positions. The spectrum of AAPBA (DMSO-d6) δ showed the following assignments: 5.71 (2H,1-H), 6.48 (1H,2-H), 6.35 (1H,3-H), 10.0 (1H,4-H) and 7.2–8.0 (the H of benzene ring). PTE showed the following assignments: ^1^H NMR (DMSO-d6): δ: 6.03–6.08 (2H, 1-H), 6.26 (1H,2-H), 3.73 (3H,4-H), 6.82 (1H, 5-H) and 7.28–7.69 (the H of benzene ring). p(AAPBA) (NaOD + D_2_O; pH = 9.5) δ showed the following assignments: 8.3 (1H-4-H), 2.23 (1H,5-H), 2.64 (2H,6-H), 2.85 (1H,7-H) and 6.68–7.8 (the H of benzene ring). And p(AAPBA-b-PTE) (NaOD + D_2_O; pH = 9.5) δ showed the following assignments: 4.75(1H,1-H), 2.64 (1H,2-H), 3.67 (6H,6-H), 2.35 (4H,7-H), 8.4 (2H,8-H) and 6.32–7.439 (the H of benzene ring). The trend of our results is nearly consistent with the research of Zhang and Liu [30, 31]. The results indicated that PTE and AAPBA were successfully polymerised.

The results of FTIR are shown in Fig. 1e. First, AAPBA has four main characteristic absorption bands, which are C=O str (1660 cm^−1^), C=C str (1640 cm^−1^), and o-b-o (1351 cm^−1^). Moreover, we can see that the benzene ring skeleton of AAPBA exists between 1555 cm^−1^ and 1610 cm^−1^, and an absorption peak of m-substituted benzene occurs at 698 cm^−1^. There are two main characteristic peaks of PTE, C=C str (1660 cm^−1^), CH_3_ str (1370 cm^−1^) and C=O str (1510 cm^−1^). In p(AAPBA-b-PTE)2, the absorption peaks of C=C of PTE and AAPBA disappeared, which proves that polymerisation was successful. Furthermore, the FTIR spectra of p(AAPBA-b-PTE)2 showed two obvious absorption peaks of PTE (1370 cm^−1^, 1510 cm^−1^), which proved that PTE was successfully embedded in the p(AAPBA-b-PTE) polymer. Compared with p(AAPBA), p(AAPBA-b-PTE)2 has an O-B-O STR absorption peak at 1330 cm^−1^ and an NH absorption peak at 1560 cm^−1^, which indirectly proves that AAPBA was successfully incorporated into the polymer. Similar to PTE, curcumin also has anti-inflammatory, anticancer, antivirus, and other pharmacological activities but also shows poor solubility and bioavailability [32]. To date, several curcumin carriers have been synthesised as drug delivery systems using viruses, liposomes, magnetic NPs, ultrasound microbubbles and so on [33, 34]. Meng [35] successfully prepared zein/carboxymethyl dextrin NPs to encapsulate curcumin. An electrostatic interaction and hydrogen bonding between zein and carboxymethyl dextrin CMD may have occurred in the formation of the composite NPs. The characteristic peaks of curcumin disappeared or transferred in the zein/CMD-cur NPs. Our results were similar. The little characteristic peaks of the PTE disappeared or shifted, which confirmed that we successfully prepared PTE as part of the NPs.

Next, we observed the TG curves (Fig. 1f) and derivative TG (DTG) curves (Fig. 1g) of p(AAPBA) and p(AAPBA-b-PTE)2. The TG results revealed that the higher the temperature was, the lower the weight of p(AAPBA-b-PTE) was. The TG curves decreased sharply between 100 °C and 350 °C, which indicated the removal of solvent molecules from the sample. The DTG results mainly showed three degradation temperatures of p(AAPBA-b-PTE)2: 290 °C, 342 °C and 404 °C. It can be seen that two degradation stages occurred at approximately 342 °C and 407 °C for p(AAPBA). The first degradation temperature of p(AAPBA-b-PTE)2 can be considered a hydrogen-bonded free group. The second degradation temperature of p(AAPBA) and p(AAPBA-b-PTE)2 was 342 °C, which may be the thermal decomposition temperature of the side chain residues. The peaks of p(AAPBA) at 407 °C and p(AAPBA-b-PTE)2 at 404 °C are the thermal degradation temperature of the main chain. These results demonstrated that p(AAPBA-b-PTE) was stable and thermolytic. The molecular weight and polydispersity index (PDI) of p(AAPBA-b-PTE) are shown in Additional file 1: Table S1. As the PTE content in p(AAPBA-b-PTE) decreased, its MW and Mn gradually increased, and the PDI remained stable.

### Performance of p(AAPBA-b-PTE) NPs

Since the pKa of PBA and its derivatives is much higher than the pH of the human body, it does not have an ideal glucose sensitivity in pH 7.5 conditions [36]. To ensure that glucose sensitivity can be maintained under normal physiological conditions, the copolymers need to reduce the pKa (the pH in the human body) to maintain a dynamic balance. Therefore, we measured the pH, temperature and glucose sensitivity of p(AAPBA-b-PTE) NPs and comprehensively considered its performance in the human physiological environment.

**Fig. 2:**
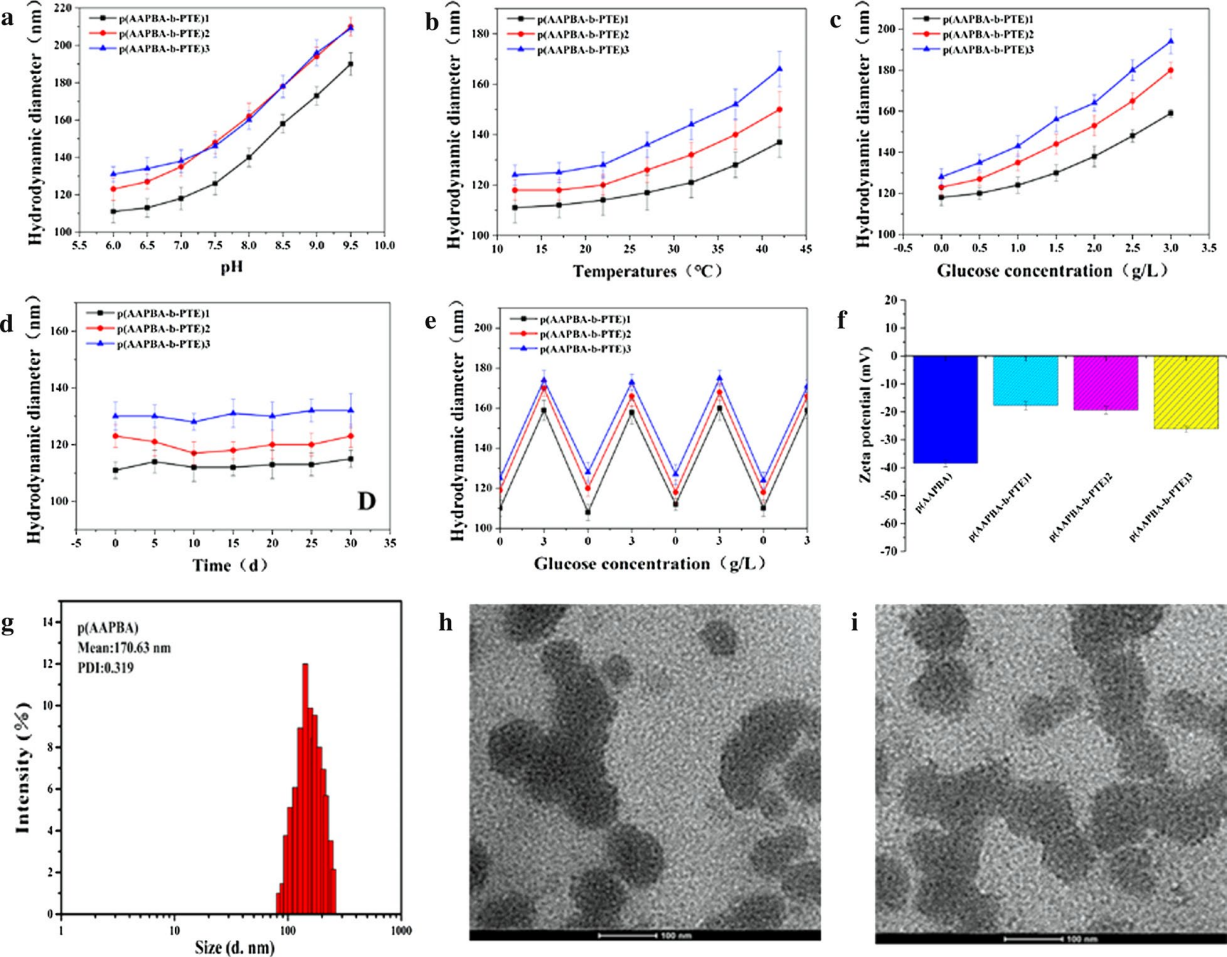
Changes in different hydrodynamic diameters: PH (**a**); temperature (**b**); glucose concentration (**c**); **d** is the p(AAPBA-b-PTE) of stability in pH 7.4 PBS. Results of glucose-sensitive elasticity of NPs. **f** Zeta potential of the p(AAPBA-b-PTE). **g** Particle size and PDI of the p(AAPBA-b-PTE). TEM change in the diagram of p(AAPBA-b-PTE)2 NPs: **h** in the PBS solution (**i**) in the 3 mg/mL glucose concentration at 72 h

Figure 2a–c revealed the results of the pH, temperature and glucose sensitivity of p(AAPBA-b-PTE) NPs. First, the size of p(AAPBA-b-PTE) NPs increased with the increase in pH, which may have been related to the pH sensitivity of AAPBA. When the pH was between 6.0 and 6.5, the particle sizes of NPs were in a relatively stable state. When the pH is greater than 6.5, phenylboronic acid groups began to appear in the AAPBA, which induces an increase in the size of NPs. It has good glucose sensitivity under physiological conditions.

Second, p(AAPBA-b-PTE) NPs are sensitive to temperature change. The particle size of p(AAPBA-b-PTE) NPs was stable at 12.5–17.5 °C. The higher the temperature, the larger the particle size. P(AAPBA-b-PTE) NPs have no temperature sensitivity, but as the temperature increases, PTE was released from p(AAPBA-b-PTE) NPs. Therefore, the NPs become hydrophilic, so the particle size increases.

Finally, p(AAPBA-b-PTE) NPs have good glucose sensitivity. When the glucose concentration was 1.5 g/L (general blood glucose value of diabetic patients), the p(AAPBA-b-PTE) NPs began to show good glucose sensitivity. It was suggested that p(AAPBA-b-PTE) NPs can intelligently release insulin in diabetic patients. Compared with our previously prepared p(NVCL-co-AAPBA) NPs [12], the addition of PTE did not damage the phenylboronic acid functional group, which had a more stable glucose sensitivity. The results of the structural stability test of the p(AAPBA-b-PTE) NPs showed that the particle size was relatively stable within 35 days of storage, and there was little difference in the particle size. It was reported that the particle size of NPs can affect drug release, cell uptake and so on, which is an important parameter for determining the drug delivery efficiency of NPs [8]. If the prepared NPs can intelligently adjust the particle size under human physiological conditions, it is easier to disperse through the barrier to various parts of the body. In terms of our results, the particle size distribution and law of change are beneficial in managing the treatment of diabetes.

It is a challenge to achieve reversible and repeatable release of glucose-sensitive drugs under specific physiological conditions. Figure 2e reveals that p(AAPBA-b-PTE) NPs have good dynamic regulation properties and reversible glucose sensitivity. When p(AAPBA-b-PTE) NPs were treated with a 3 g/L concentration of glucose, they swelled and the particle size gradually increased. Then, when placed in 0 g/L glucose concentration, the size of p(AAPBA-b-PTE) NPs was significantly reduced, close to their original size. After repeated testing, the results were consistent. This suggests that the prepared NPs can adapt to different concentrations of blood glucose by improving the particle size. The trend of the results is consistent with the characteristics of NPs prepared by Wu [13]. In the presence of glucose, more phenylboronic acid groups are transformed from hydrophobic non-ionic groups into hydrophilic, negatively charged phenyl borate esters, so the swelling degree of NPs increased. The p(AAPBA-b-PTE) NPs exhibited an obvious glucose response after the introduction of phenylboronic acid groups.

Next, we observed the size of p(AAPBA-b-PTE) NPs using DLS and analysed its stability based on the zeta potential. The results are shown in Fig. 2f–g. The size of the NPs was approximately 170 nm and its zeta potential was negative. The circulation of the nano-drug delivery system with a negative charge on the surface is expected to last longer in the blood. As the PTE content in p(AAPBA-b-PTE) NPs decreases, the zeta potential increases and its absolute value decreases. The lower that the absolute value of the zeta potential is, the more likely it is to condense. This indicated that the PTE stabilised and dispersed the p(AAPBA-b-PTE) NPs. In addition, the distribution of the PDI did not change significantly. It was confirmed that the p(AAPBA-b-PTE) NPs were uniformly distributed and exhibited good dispersion stability.

Figure 2h–i display a TEM diagram of the p(AAPBA-b-PTE) NPs. NPs are spherical, but exhibit aggregation adhesion. Compared with that in the phosphate-buffered saline (PBS) solution, the NPs containing insulin appeared as large pieces of fusion in the glucose solution, the particle size was obviously enlarged and the distribution gradually broadened. This indicated that the NPs containing insulin could effectively decompose and release insulin in the glucose solution. Guo [37] et al. synthesised an amphiphilic block sugar copolymer (P[AAPBA-b-GAMA]) from phenylboronic acid and carbohydrates. It was spherical with good dispersibility. Ayubi [38] et al. modified the surface of magnetic NPs (MNP@PEG-Cur) with pegylated curcumin, and the NPs displayed aggregation and adhesion. The p(AAPBA-b-PTE) NPs also exhibited adhesion and poor dispersion. It may be that the esterified PTE combined with AAPBA facilitates swelling and aggregates in the glucose solution.

Due to the great interest of researchers in the drug product development of NPs, more methods are needed to evaluate the quality, safety and efficacy of NPs. Li [39] summarised the pharmacokinetic modelling and simulation methods based on physiology to describe and predict the absorption, distribution, metabolism and excretion of NPs in vivo. The degradation process of p(AAPBA-b-PTE) NPs was observed by TEM. Additional file 1: Fig. S1 shows that p(AAPBA-b-PTE) NPs completely degraded within 40 days. First, the NPs began swelling on day one and then continued to spread outward. After three days, a reticular structure and dilution were observed. After 10 days, only a few NPs had not been degraded whereas the rest had dissolved. After 40 days, the NPs were completely degraded under a 500 nm microscope. The results also indirectly confirmed that p(AAPBA-b-PTE) NPs could be effectively degraded in the human physiological environment. Zhang [40] et al. prepared a multifunctional microgel by using the precipitation emulsion method with *N*-isopropyl acrylamide (NIPAAm), ethyl methacrylate (2-dimethylamino) methacrylate (DMAEMA) and AAPBA, and they gradually degrade with time (the specific complete degradation time was not reported). In the physiological environment of the human body, PTE releases its own pharmacological activity, which causes the structure of p(AAPBA-b-PTE) NPs to dissolve and become absorbed in the body.

### Insulin loading and release of PTE

**Fig. 3:**
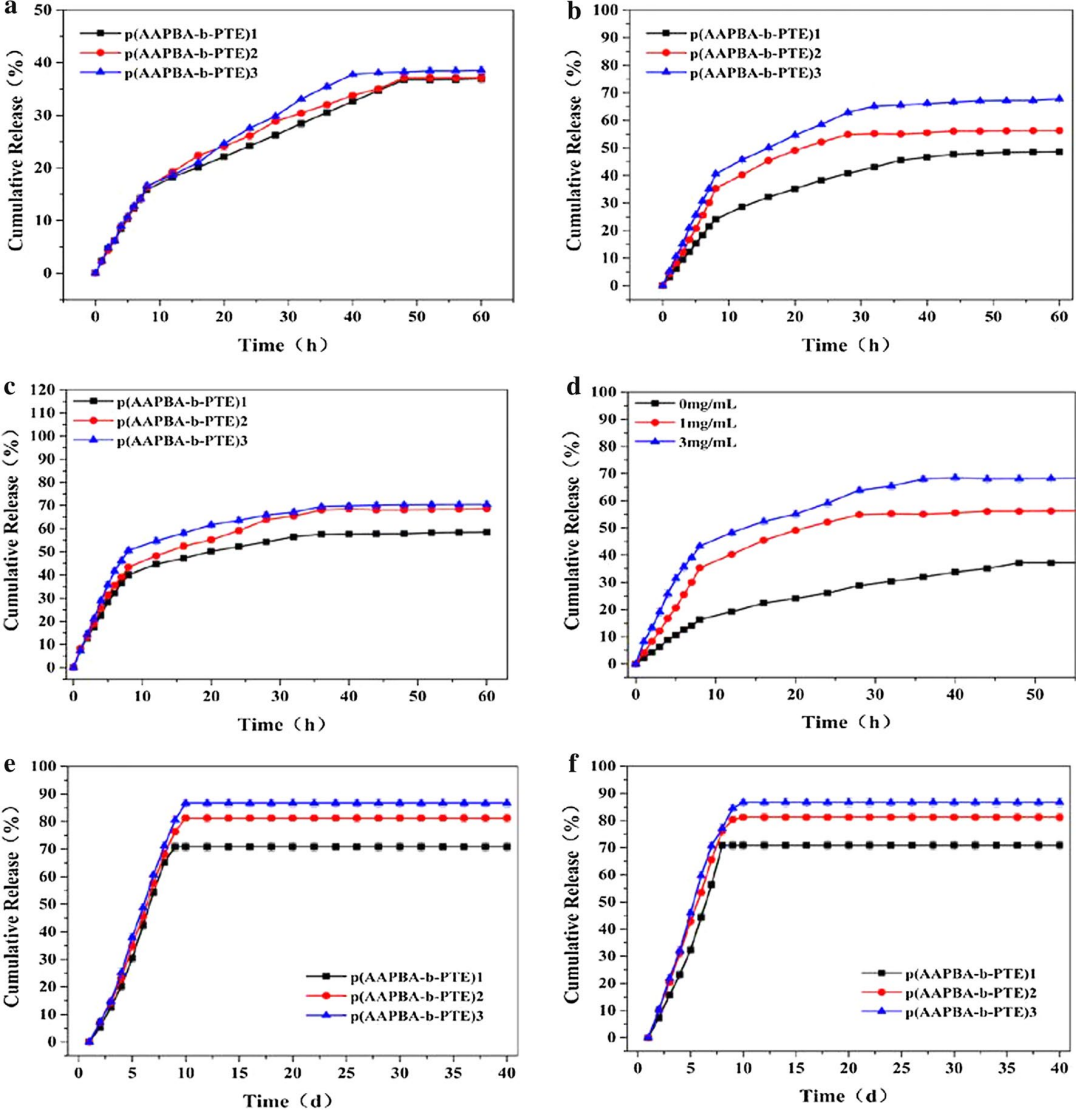
The cumulative insulin release of p(AAPBA-b-PTE) NPs in vitro with glucose concentration of 0 mg/ml (**a**), 1 mg/ml (**b**) and 3 mg/ml (**c**). **d** is the cumulative insulin release of p(AAPBA-b-PTE)2. The cumulative PTE release of p(AAPBA-b-PTE) NPs in vitro with glucose concentration of 0 mg/ml (**e**) and 3 mg/ml (**f**)

The performance test of p(AAPBA-b-PTE) NPs determined their capability to encapsulate insulin in NPs and intelligently release the insulin in diabetic patients. The results in Table 2 show that the EE of insulin-loaded p(AAPBA-b-PTE) NPs was approximately 56%, and the LC was approximately 13%. Moreover, the EE and LC exhibited little fluctuation and remained stable. However, the EE of insulin NPs prepared with chitosan and water-soluble snail mucin by Mumuni MA [41] et al. was 88.6%. Chen [42] prepared spherical NPs from six-armed star-shaped poly(lactide-co-glycolide)(6-s-PLGA) NPs that were used to encapsulate puerarin (PU-NPs). Its EE was 89.52 ± 1.74%, and the LC was 42.97 ± 1.58%. It is speculated that the reason for the relatively low EE of p(AAPBA-b-PTE) NPs is that insulin is a hydrophilic drug, which means that it easily enters the outer water phase from the organic phase. Overall, the PTE formed a more compact complex structure with AAPBA. Figure 3a–e show the insulin release characteristics of p(AAPBA-b-PTE) NPs. Next, the release rule of insulin from p(AAPBA-b-PTE) NPs was analysed. At 1 ml and 3 ml glucose concentrations, insulin achieved a rapid release within 10 h and remained stable after 30 h, with a release amount up to 70% (Fig. 3b, c). The insulin release of p(AAPBA-b-PTE)2 increased as the glucose concentration increased. The release trend of p(AAPBA-b-PTE)2 was similar to that of p(AAPBA-b-PTE)1, and it had a suitable release amount. From the perspective of environmental protection and performance, we considered p(AAPBA-b-PTE)2 for related research. Meanwhile, the circular dichroism (CD) spectrum was detected by BRIGHT TIME Chirascan (Jasco-815, UK Applied Optophysics). The CD spectra results (Additional file 1: Fig. S2) concluded that the structure of insulin in NPs was not destroyed. The CD spectrum of insulin released from NPs was nearly consistent with that of standard insulin. Insulin could be effectively encapsulated in p(AAPBA-b-PTE) NPs. This also indirectly illustrated that the hypoglycaemic effect of insulin was not impaired by its encapsulation in the NPs.

**Table 2:** Insulin LC and EE of p(AAPBA-b-PTE) NPs

Samples	Insulin concentration (mg/mL)	EE (%)	LC (%)
p(AAPBA-b-PTE)1	1.00	55.1 ± 5.4	13.3 ± 1.6
p(AAPBA-b-PTE)2	1.00	56.7 ± 6.1	13.8 ± 1.4
p(AAPBA-b-PTE)3	1.00	58.3 ± 5.1	14.5 ± 1.2

Data are presented as mean ± SD

*EE* encapsulation efficiency, *LC* drug loading

PTE, as a plant extract, reduces blood glucose and can be used to treat diabetes complications. The pharmacokinetics of PTE has been confirmed in animals and humans [43]. We examined the pharmacological activity and release pattern of PTE from p(AAPBA-b-PTE) NPs under the glucose concentration. The results show that p(AAPBA-b-PTE) NPs could effectively release PTE (Fig. 3e, f). The PTE maintained a release amount of over 70% after 10 days and tended to remain stable. This indicated that most of the pharmacological activities of PTE were present in the p(AAPBA-b-PTE) NPs. PTE has been released efficiently and persistently in p(AAPBA-b-PTE) NPs, which indicates their superior role in the prevention and treatment of diabetes complications in animal experiments. Sanna [44] et al. developed a novel cationic chitosan and anionic alginate (ALG)-coated poly (D, l-lactide-glycolide) NPs loaded with bioactive polyphenol trans-(E)-resveratrol (RSV). PTE, which is a derivative of RSV and a polyphenolic compound, has similar biological activities. The NPs encapsulated with RSV exhibited a first burst release of RSV (approximately 70%) within 30 min, followed by a slow release of RSV within 6 h. Although the first burst release of p(AAPBA-b-PTE) NPs occurred within 10 days, we achieved a long-lasting, effective release within 40 days, which had long-term therapeutic effects on diabetes.

After that, we used Origin software to perform the Ritger–Peppas equation to quickly fit the curve of the drug release law of the carrier drug delivery system. It can be seen in Additional file 1: Table S2 that p(AAPBA-b- PTE)2 had N values ranging from 1 to 5 and R2 values higher than 0.9 at glucose concentrations of 0, 1 and 3 mg/ml. k was greater than 0.89, and the transmission mechanism was non-Fickian diffusion. These results mean that the drug release mechanism was skeleton dissolution, and the drug release was relatively complete, which can be used in a polymer drug release system with many potential applications.

### Toxicological research

**Fig. 4:**
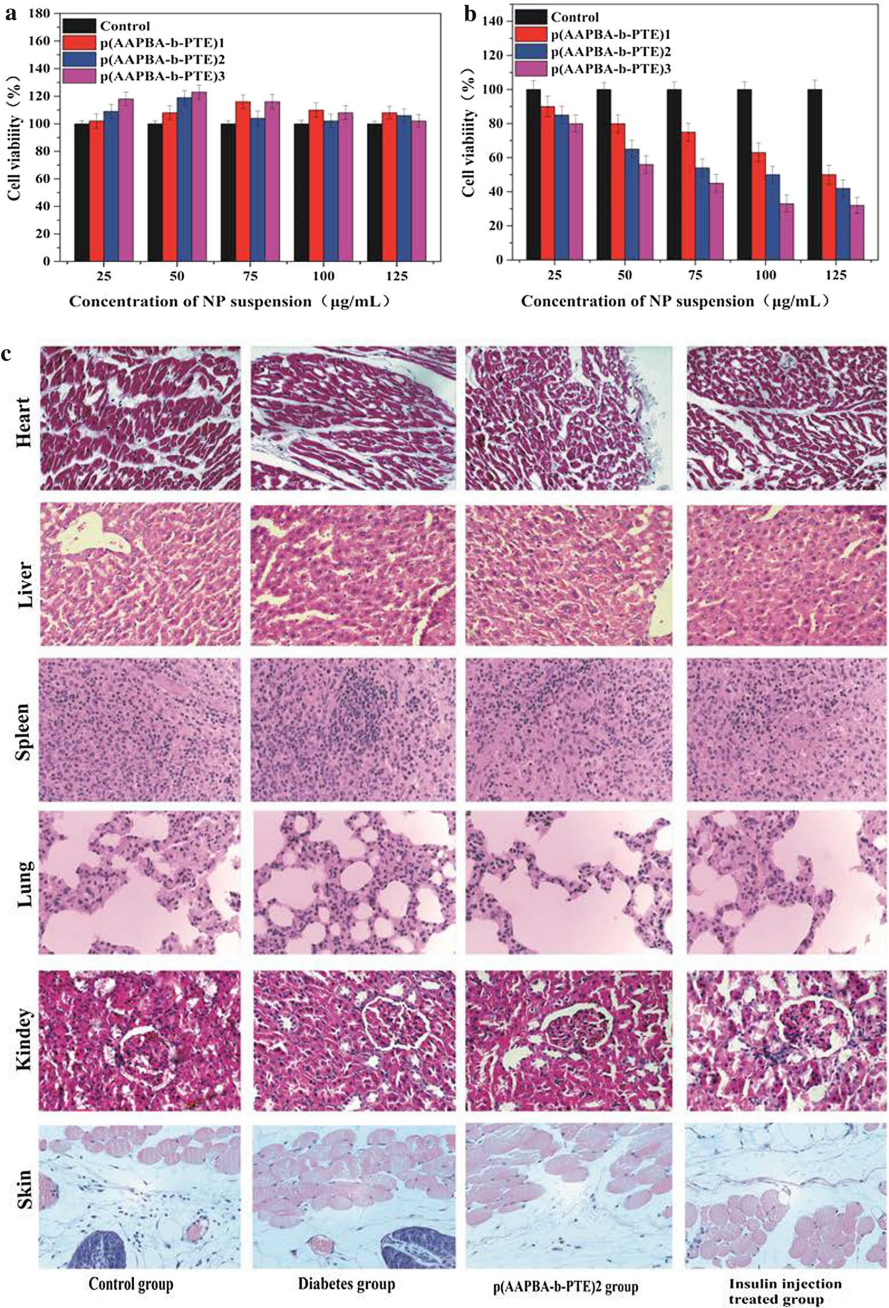
**a**, **b** Cell viability as a function of the concentration of p(AAPBA-b-PTE) NPs by the MTT assay at 37 °C after 24-h the incubation. Each value represents the mean ± SD (n = 5). **c** HE stained images representative images of the heart, liver, spleen, lung, kidney and skin of the control group, diabetic group, p(AAPBA-b-PTE)2 group and insulin injection treated group

The process of treating diabetes is long; therefore, the drug carrier used for treating diabetes must be safe, nontoxic and biodegradable so that it will not cause secondary damage to the human body [45]. Thus, we continued to explore the toxicity and adverse side effects of p(AAPBA-b-PTE) NPs and compared the differences between NPs prepared at different ratios. First, we evaluated the NPs through cell experiments. A cell culture is sensitive to environmental changes and can sensitively detect the existence of potentially toxic substances. We selected human normal liver L0_2_ cells and human tumour SMMC-7721 cells for comparison. Non-pretreated cells were used as a negative control group. All cells were exposed to suspensions of different concentrations (25–125 µg/mL). The results shown in Fig. 4a revealed that the cell viability of human normal liver L0_2_ cells remained above 100% after treatment with different concentrations of suspensions and different NPs. The existence of p(AAPBA-b-PTE) NPs will not damage the survival rate of human normal liver L0_2_ cells. However, the cell viability of human liver cancer SMMC-7721 cells decreased to varying degrees after treatment with NPs, with the lowest being 30%. The greater the PTE content in p(AAPBA-b-PTE) NPs, the higher the cell survival rate was. The pharmacological activity of PTE reduces the toxicity of biomaterials, thus reducing the lethality of cells. Zhou [46] et al. prepared NPs (GLPNPs) by boiling purified liquorice proteins in an aqueous solution. They employed L0_2_, MDCK, HepG2, and Caco-2 cell lines, respectively, for cytotoxicity evaluation. However, self-assembly into nanostructures did not significantly alter the cytotoxicity of GLP proteins. Compared with the p(AAPBA-b-OVZG) NPs previously prepared with low toxicity [13], the toxicity study of p(AAPBA-b-PTE) NPs achieved a breakthrough. Table 3 shows that the p(AAPBA-b-PTE) NPs did not significantly affect the blood biochemical indexes of mice compared with those of the normal group. Lin [47] et al. observed subacute effects of low-dose combined poisoning with silica NPs (SiNPs) and lead acetate (PB) on the cardiovascular system of SD rats through routine blood testing and blood biochemical analysis. p(AAPBA-b-PTE) NPs were indirectly demonstrated to be safe, nontoxic and suitable for long-term prevention and treatment of diabetes.

**Table 3:** Effect of administration by injection of the NPs on the biochemical parameters of rats after 14 d (n = 5, mean ± SD)

Index/groups	Normal	P(AAPBA-b-PTE)2		
		10 mg/kg/d	50 mg/kg/d	100 mg/kg/d
RBC (× 10^6^ µl^−1^)	10.42 ± 0.45	10.35 ± 0.68	10.38 ± 0.46	10.51 ± 0.34
MCV (fL)	41.56 ± 1.06	40.89 ± 0.98	41.23 ± 1.14	41.68 ± 1.14
RDW(%CV)	17.95 ± 2.54	18.01 ± 2.34	18.12 ± 2.18	18.24 ± 2.21
HCT (vol.%)	43.0 3 ± 2.01	42.80 ± 1.86	42.95 ± 2.04	43.01 ± 1.95
MCH (pg)	13.85 ± 0.86	13.80 ± 0.78	13.92 ± 0.65	13.95 ± 0.62
MCHC (g/L)	33.40 ± 1.53	33.80 ± 1.68	33.50 ± 1.48	33.62 ± 1.54
WBC (× 10^3^ µl^−1^)	7.95 ± 1.25	7.80 ± 1.15	8.02 ± 0.95	7.90 ± 1.20
NE (%)	18.22 ± 2.57	18.24 ± 2.38	18.19 ± 2.26	18.12 ± 2.12
EO (%)	2.72 ± 1.18	2.70 ± 1.22	2.74 ± 1.12	2.70 ± 1.20
BA (%)	1.80 ± 0.64	1.79 ± 0.58	1.81 ± 0.70	1.78 ± 0.54
LY (%)	74.01 ± 3.09	74.31 ± 2.85	73.69 ± 2.92	74.19 ± 2.68
MO (%)	2.38 ± 0.58	2.40 ± 0.37	2.35 ± 0.65	2.34 ± 0.50
PLT (10^3^/mm^3^)	768.20 ± 150.80	770.35 ± 145.54	776.25 ± 153.40	765.38 ± 148.26
MPV (fL)	6.01 ± 1.78	5.98 ± 1.69	6.04 ± 1.70	6.03 ± 0.26

The results of mouse histomorphology also confirmed the safety of NPs. It can be seen from Fig. 4c that, compared with the control group, the fat vacuoles in the liver of the diabetic group increased; the liver was damaged to some extent; and the remaining tissues were almost unchanged. The mechanism of diabetes was mostly related to heredity and abnormal glucose and lipid metabolism, which are also causes of liver damage [48]. Compared with the control group, there was no significant difference in the heart, liver, spleen, lung and kidney of the group injected with p(AAPBA-b-PTE)2 NPs, which further proved that NPs are safe and harmless. Compared with the diabetic group, the damage to tissues and organs was relatively reduced and was protected from hyperglycaemia to some extent. In a chronic exposure environment, only a limited number of studies have focused on evaluating the effects of inorganic NPs on organ toxicity, inflammation, immunotoxicity and genotoxicity [49]. At present, the long-term toxicity of NPs has yet to be verified [50]. As far as our results are concerned, PTE showed a good trend of lessening the toxicity of NPs.

It is worth noting that there are obvious bleeding spots after subcutaneous injection of NPs in mice. The skin tissue layer of the p(AAPBA-b-PTE)2 NPs group was clear, with an orderly arrangement of epidermal cells and no obvious degeneration or necrosis of the epidermal cells. However, a small amount of vasodilation and congestion, interstitial oedema and inflammatory cell infiltration can be seen in the dermis, whereas the hair follicle structure, sebaceous glands and other skin appendages appeared normal. The current major challenge of oral insulin is to overcome the multiple barriers of the gastrointestinal tract. Different insulin polysaccharide NPs were used to protect insulin from becoming chemically and enzymatically degraded in the stomach and small intestine, promote mucus permeation and, finally, achieve sustained hypoglycaemic effects [51]. Therefore, NPs encapsulated with insulin usually enter the body through intravenous injection. However, we selected subcutaneous injection to make it possible to observe the mice skin pathology. It may be that the relatively large size of NPs contributes to the occurrence of bleeding points. NPs are difficult to absorb subcutaneously, and capillaries have a certain resistance to NPs. In our next work, we will attempt to further improve the preparation method of NPs so that it is closer to the human body barrier and can achieve a sustained, more effective release.

### Evaluation of hypoglycaemic characteristics, oxidation and micro-inflammation in vivo

**Fig. 5:**
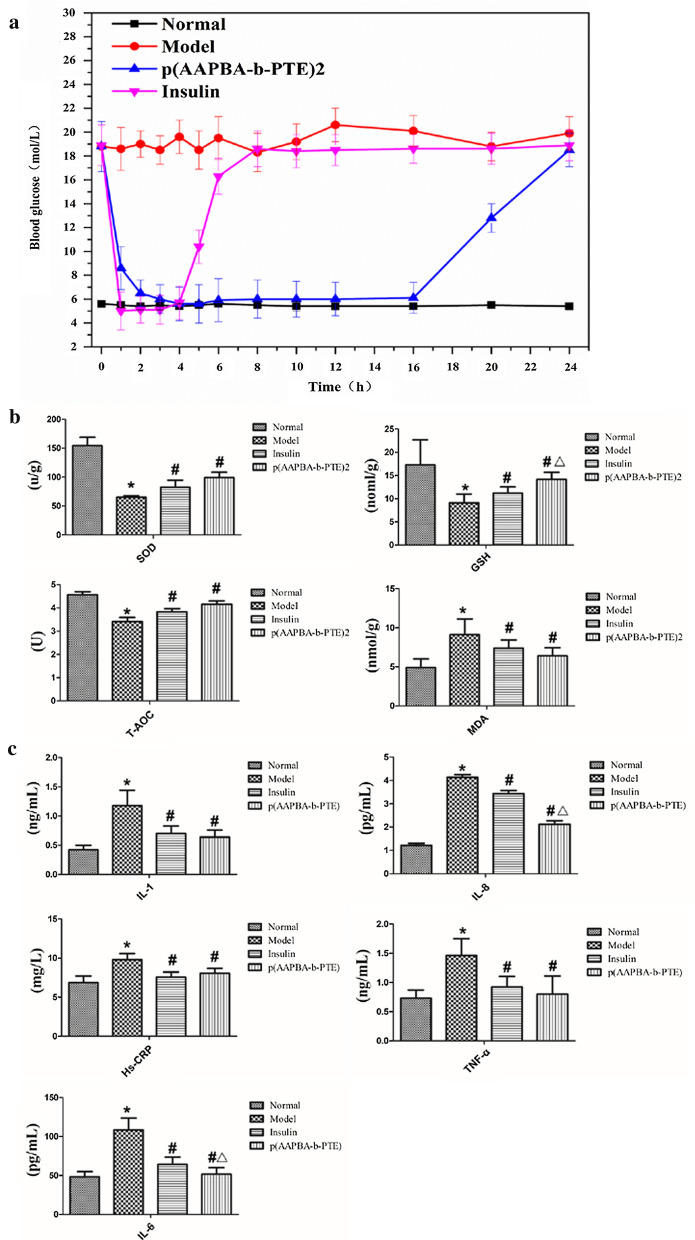
**a** Blood glucose concentration after injection over 24 h. **b** Oxidation index and **c** microinflammatory index of mice in each group after 2 weeks

Under the condition of confirming that p(AAPBA-b-PTE) NPs are safe and harmless, we further discussed the hypoglycaemic effect of insulin-loaded NPs in vivo. From the blood glucose curves, it can be seen that both the control group and diabetic group remained within a stable curve range throughout the experiment. The p(AAPBA-b-PTE)2 group and insulin injection treated group exerted an obvious hypoglycaemic effect. However, the tendency to lower blood glucose was different for these two groups. It can be seen from Fig. 5a that the blood glucose in the insulin injection treated group was stabilised at 5–6 mmol/L within 4 h, but then showed a straight upward trend, after which it tended to be stable after 8 h. In the p(AAPBA-b-PTE)2 group, the blood glucose was maintained at 5–6 mmol/L within 16 h. P(AAPBA-b-PTE)2 NPs have been proven to have a hypoglycaemic effect in vivo and can stably reduce blood sugar within 16 h. Taili [52] synthesised carbon-NPs (CNPs) through the hydrothermal treatment of polysaccharides obtained from *Arctium lappa L*. They investigated the hypoglycaemic effect of CNPs on a high-fat diet plus streptozotocin-induced diabetic mice. The results showed that CNPs reduced fasting blood glucose in mice after 42 days. Relatively speaking, the NPs constructed based on AAPBA had a remarkable and rapid effect of reducing blood glucose. Furthermore, due to the addition of PTE, the hypoglycaemic effect was more obvious and long-lasting.

SOD, which directly reflects the antioxidation level of the body. MDA is also a sign of oxidative stress and is the final product of lipid peroxidation [53]. From Fig. 5b, we found that p(AAPBA-b-PTE)2 NPs containing insulin can up-regulate SOD activity and decrease MDA levels in diabetic mice. Compared with the insulin injection group, the GSH of the p(AAPBA-b-PTE)2 NPs group significantly increased (*p* < 0.05). P(AAPBA-b-PTE)2 NPs containing insulin improved the antioxidant capacity and micro inflammation status of diabetic mice by releasing the pharmacological activity of PTE, which indirectly proved that the plant extracts possessed a pharmacological effect in polymer sustained-release materials.

Insulin resistance is the main pathophysiological feature of diabetes, and inflammation plays an important role in the occurrence and development of insulin resistance through various cytokines and molecular pathways. It has been reported [54] that increases of inflammatory cytokines, such as TNF-α and IL-6, are related to the severity of diabetes. Hs-CRP is not only a nonspecific marker of inflammation but is also directly involved in cardiovascular diseases, such as inflammation and atherosclerosis, which is the most powerful predictor and risk factor of cardiovascular diseases [55]. Figure 5c shows that the levels of IL-6 and TNF-α in mice after treatment with p(AAPBA-b-PTE)2 NPs containing insulin for two weeks were significantly lower than those of the model group (*p* < 0.05). There was no significant difference in the CRP level, but there was a downward trend. Our results suggest that p(AAPBA-b-PTE)2 NPs can reduce the levels of TNFα, IL-6 and Hs-CRP in diabetic rats and improve the inflammatory status. Pure use of biomaterials without plant extracts, such as those used in the study by Zhang [40], which were prepared in a multifunctional microgel from N-isopropyl acrylamide (NIPAAm), 2-dimethylaminoethyl methacrylate (DMAEMA) and AAPBA using a precipitation emulsion method, can reduce blood glucose but has no therapeutic effect for treating diabetes complications. The cross combination of biochemical with plant extracts pioneers a new field.

## Conclusions

In this study, PTE was used as part of a polymeric material to prepare NPs, which reduced the toxicity of AAPBA biomaterials while also improved the poor water solubility and low bioavailability of PTE. PTE has higher stability and more sustained release characteristics in NPs. Moreover, the p(AAPBA-b-PTE) NPs fully exerted the pharmacological activity of PTE, effectively lowered blood glucose, improved the antioxidant capacity and reduced the inflammatory response. NPs prepared with the glucose-sensitive material AAPBA provide a ‘switch’ for insulin release in diabetic patients. PTE expands its application in the treatment of diabetes through NPs or other drug delivery media. In addition, plant extracts, which possess similar activities to ginseng extract, tea polyphenols, theanine and anthocyanins, play an important role in improving cardiovascular function, anticancer, prevention and treatment of diabetes and the immune system. We predict that a nano delivery system utilising plant extracts will provide new capabilities for future therapeutic applications.

## Supplementary Information

**Additional file 1: Figure S1.** Nanoparticle swelling images under TEM after 1(a), 4(b), 10(c), 40(d)days.** Figure S2.** CD spectra of the insulin released from NPs and standard insulin.** Table S1.** Comparison with other systems.** Table S2.** The Mw and Mn and PDI of the p(AAPBA-b-PTE).** Table S3.** Ritger–Peppas results of p(AAPBA-b-PTE)2 NPs.

## Data Availability

All data generated or analysed during this study are included in this published article.

## Abbreviations

PTE: Pterostilbene
AAPBA: 3-acrylamidophenyl boric acid
NPs: Nanoparticles
p(AAPBA-b-PTE): Poly(3-acrylamidophenyl boric acid-b-pterostilbene)
