# Supplementary material for: Nanoparticles prepared from pterostilbene reduce blood glucose and improve diabetes complications
Source: J Nanobiotechnology. 2021 Jun 27;19:191. doi: 10.1186/s12951-021-00928-y (PMC8237509; doi:10.1186/s12951-021-00928-y)
Supplement: Supplementary file 1 — Additional file 1: Figure S1. Nanoparticle swelling images under TEM after 1(a), 4(b), 10(c), 40(d)days. Figure S2. CD spectra of the insulin released from NPs and standard insulin. Table S1. Comparison with other systems. Table S2. The Mw and Mn and PDI of the p(AAPBA-b-PTE). Table S3. Ritger–Peppas results of p(AAPBA-b-PTE)2 NPs. [file 12951_2021_928_MOESM1_ESM.docx]

**Nanoparticles prepared from pterostilbene reduce blood glucose and improve diabetes complications**

Xi Zhao ^a1^, Anhua Shi ^a1^, Qiong Ma ^a^, Xueyan Yan^b^, Ligong Bian^c^, Pengyue Zhang ^d*^, Junzi Wu ^a *^

^a^ Yunnan Provincial Key Laboratory of Molecular Biology for Sinomedicine, School of Basic Medical, Yunnan University of Chinese Medicine, Kunming, Yunnan 650500, P.R. China

^b^ College of Second Clinical Medicine, Kunming Medical University, Kunming, Yunnan 650500, PR China

^c^ College of Basic Medicine, Kunming Medical University, Kunming, Yunnan 650500, PR China

^d^ Key Laboratory of acupuncture and Tuina for Treatment of Encephalopathy, College of Acupuncture, Tuina and Rehabilitation, Yunnan University of Traditional Chinese Medicine, Kunming, 650500, China;

^1^ These authors contributed equally to this work.

*Corresponding authors: E-mail: [xnfz@ynutcm.edu.cn](mailto:xnfz@ynutcm.edu.cn%20) (Junzi Wu); zpy19802000@163.com (Pengyue Zhang)

**The file includes:** Figure S1-S2, Table S1, Table S2, Table S3


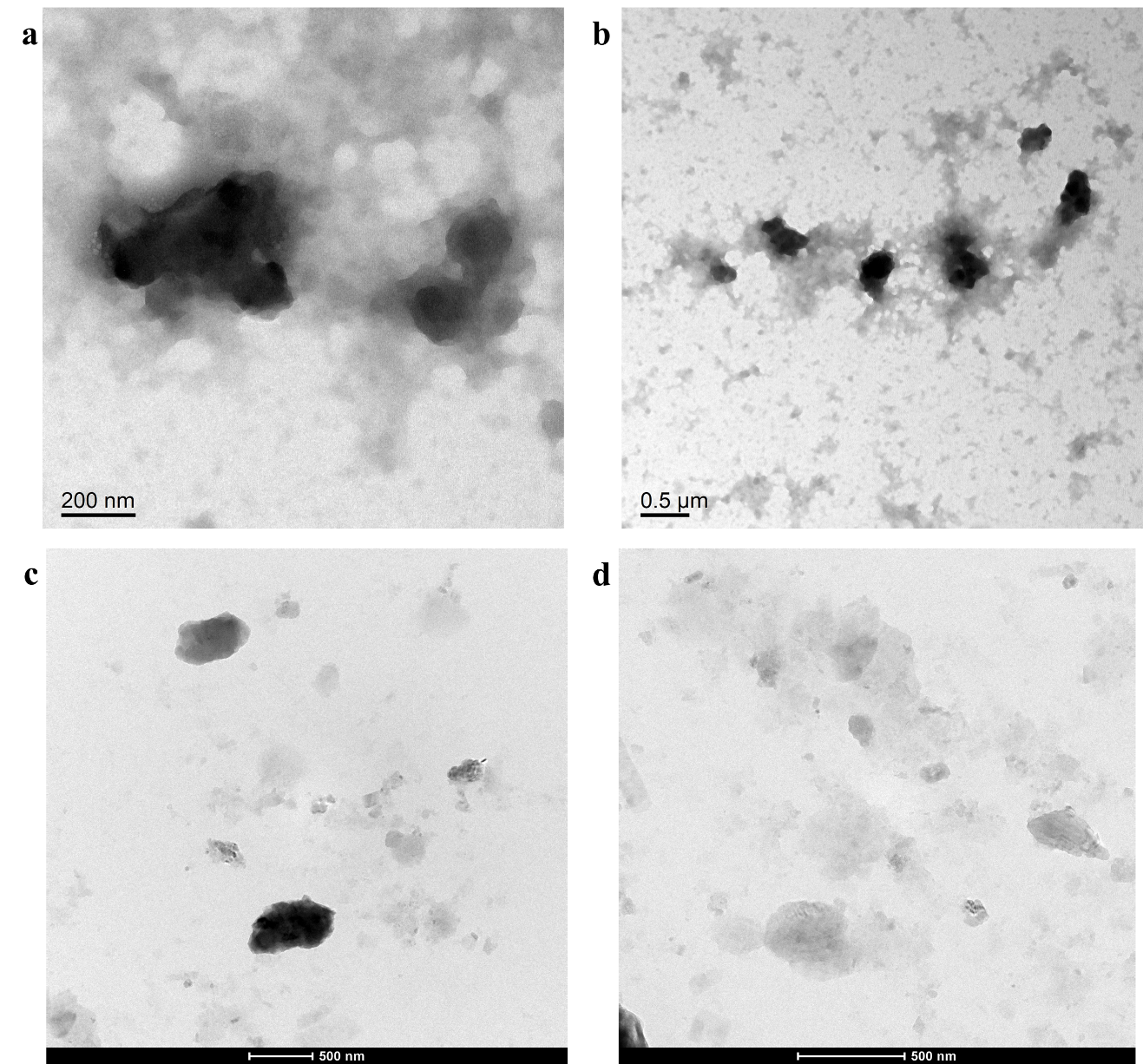


**Figure S1.** Nanoparticle swelling images under TEM after 1(**a**), 4 (**b**), 10 (**c**), 40 (**d**)days.


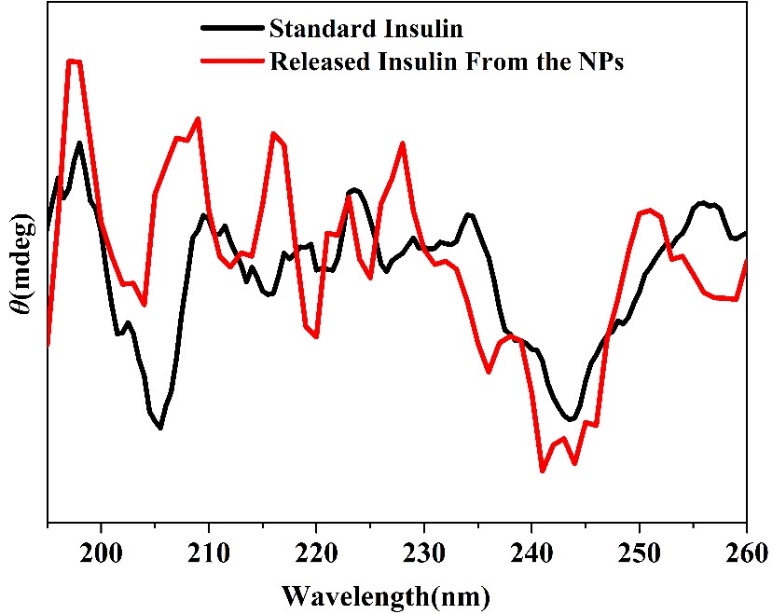


**Figure S2.** CD spectra of insulin released from NPs and standard insulin.

**Table S1.** Comparison with other systems.

|  | Typical representative | Examples of combination with AAPBA | Administration mode | Effect |
| --- | --- | --- | --- | --- |
| Thermosensitive monomers | Poly(N-isopropylacrylamide) (PNIPAM) | Poly(N-isopropylacrylamide-co-3-acrylamidophenylboronic  acid) (p[NIPAM-AAPBA])[1] | Injection | 1. Avoids toxic organic solvents and reduces side effects in the human body.  2. It can release hydrophilic and lipophilic drugs (such as insulin) simultaneously, as well as show the drug release characteristics of specific components.  3. Large amount of drug release and short time of drug release. |
|  | Diethylene glycol methyl ether methacrylate (DEGMA) | Poly(3-acrylamidophenyl boronic acid-b-diethylene glycol  methyl ether methacrylate)(p[AAPBA-b-DEGMA])[2] |  |  |
|  | N-vinylcaprolactam (NVCL) | Poly(N-vinylcaprolactam-co-acrylamidophenylboronic acid)(p[NVCL-co-AAPBA])[3] |  |  |
| pH sensitive monomers | Acrylates | Poly(ethylene glycol)-b-ploy(acrylic acid-co-acrylamidophenylboronic acid) (PEG-b-P[AA-co-AAPBA])[4] | Injection | 1. It has better glucose response and self-control of insulin release under normal human physiological pH conditions.  2. It has a certain level of toxicity. |
|  |  | Poly(acrylamide-co-3-acrylamido phenylboronic acid-co-chitosan grafted maleic acid) (P[AM-co-AAPBA-co-CSMA]s)[5] |  |  |
|  |  | Poly(N-acryloyl morpholine-block-(N-acryloyl morpholine-copentafluoroacrylate) (p[AMP-b-(AMP-co-PFPA)])[6] |  |  |
| Glycolipid monomers | D-gluconamidoethyl methacrylate (GAMA) | Poly(D-gluconamidoethyl methacrylate-  block-3-acrylamidophenylboronic acid) (p[AAPBA-b-GAMA])[7] | Injection | 1. Low toxicity.  2. Improves the glucose response of the polymer.  3. It improves the hydrophilicity and biocompatibility of phenylboric acid.  4. Nasal administration prevents degradation of proteins and peptides in the gastrointestinal tract and metabolism of liver enzymes. |
|  | O–vinylazeloyl-D-galactose (OVZG) | Poly(3-acrylamidophenylboronic acid-b-6-O–vinylazeloyl-D-galactose) (p[AAPBA-b-OVZG])[8] |  |  |
|  | 2-lactobionamidoethyl methacrylate (LAMA) | poly(2-lactobionamidoethyl methacrylate-random-3-acrylamidophenylboronic acid) (p[LAMA-r-AAPBA])[9] | Nasogastric administration |  |
| Plant extracts | Pterostilbene (PTE) | Poly(3-acrylamidophenyl boric acid-b- pterostilbene)(p[AAPBA-b-PTE]) | Subcutaneous injection | 1. Low toxicity.  2. Releases the pharmacological activity of plant extracts to treat diseases and reduce biological toxicity.  3. It maintains good release and drug loading capacity in a normal physiological environment in humans. |

**References:**

1. Luo Q, Liu P, Guan Y, Zhang Y. Thermally induced phase transition of glucose-sensitive core-shell microgels. *ACS Appl Mater Interfaces.* 2010 Mar;2(3):760-7.

2. Wu JZ, Williams GR, Li HY, Wang D, Wu H, Li SD, Zhu LM. Glucose- and temperature-sensitive nanoparticles for insulin delivery. *Int J Nanomedicine.* 2017 May 29;12:4037-4057.

3. Wu JZ, Bremner DH, Li HY, Sun XZ, Zhu LM. Synthesis and evaluation of temperature- and glucose-sensitive nanoparticles based on phenylboronic acid and N-vinylcaprolactam for insulin delivery. *Mater Sci Eng C Mater Biol Appl.* 2016 Dec 1;69:1026-35.

4. Ma R , Wang B , Sun P , [Shi](https://xueshu.baidu.com/s?wd=author%3A%28L%20Shi%29%20&tn=SE_baiduxueshu_c1gjeupa&ie=utf-8&sc_f_para=sc_hilight%3Dperson) L. B 3Q MAS NMR Study on Glucose‐Responsive Micelles Self‐assembled from PEG‐b‐P(AA‐co‐AAPBA)[J]. *Chinese Journal of Chemistry*, 2014, 32(1):97-102.

5. Elshaarani T, Yu H, Wang L, Feng J, Li C, Zhou W, Khan A, Usman M, Amin BU, Khan R. Chitosan reinforced hydrogels with swelling-shrinking behaviors in response to glucose concentration. *Int J Biol Macromol.* 2020 Oct 15;161:109-121.

6. Gaballa H, Theato P. Glucose-Responsive Polymeric Micelles via Boronic Acid-Diol Complexation for Insulin Delivery at Neutral pH. *Biomacromolecules.* 2019 Feb 11;20(2):871-881.

7. Guo Q, Wu Z, Zhang X, Sun L, Li C. Phenylboronate-diol crosslinked glycopolymeric nanocarriers for insulin delivery at physiological pH. *Soft Matter.* 2014 Feb 14;10(6):911-20.

8.Wu JZ, Bremner DH, Li HY, Niu SW, Li SD, Zhu LM. Phenylboronic acid-diol crosslinked 6-O-vinylazeloyl-d-galactose nanocarriers for insulin delivery. *Mater Sci Eng C Mater Biol Appl.* 2017 Jul 1;76:845-855.

9. Zheng C, Guo Q, Wu Z, Sun L, Zhang Z, Li C, Zhang X. Amphiphilic glycopolymer nanoparticles as vehicles for nasal delivery of peptides and proteins. *Eur J Pharm Sci.* 2013 Jul 16;49(4):474-82.

**Table S2.** The Mw and Mn and PDI of the p(AAPBA-b-PTE).

| Samples | PTE/p(AAPBA)  mass ratio | Mw | Mn | PDI |
| --- | --- | --- | --- | --- |
| p(AAPBA-b-PTE)1 | 500:1000 | 5.7 × 10^4^ | 4.4 × 10^4^ | 1.03 |
| p(AAPBA-b-PTE)2 | 100:1000 | 5.9 × 10^4^ | 4.9 × 10^4^ | 1.10 |
| p(AAPBA-b-PTE)3 | 50:1000 | 6.4 × 10^4^ | 5.1 × 10^4^ | 1.25 |

Abbreviations: PDI is polydispersity index.

**Table S3.** Ritger–Peppas results of p(AAPBA-b-PTE)2 NPs.

| Samples | Glucose concentration（mg/mL） | Ritger–Peppas model | | | Transport mechanism |
| --- | --- | --- | --- | --- | --- |
| p(AAPBA-b-PTE)2 |  | n | k | R2 |  |
|  | 0 | 1.246 | 1.658 | 0.944 | Non-Fickian diffusion |
|  | 1 | 2.113 | 3.385 | 0.938 | Non-Fickian diffusion |
|  | 3 | 5.46 | 5.49 | 0.98 | Non-Fickian diffusion |
